# HIF-2α-induced chemokines stimulate motility of fibroblast-like synoviocytes and chondrocytes into the cartilage-pannus interface in experimental rheumatoid arthritis mouse models

**DOI:** 10.1186/s13075-015-0816-x

**Published:** 2015-10-29

**Authors:** Yun Hyun Huh, Gyuseok Lee, Keun-Bae Lee, Jeong-Tae Koh, Jang-Soo Chun, Je-Hwang Ryu

**Affiliations:** BioImaging and Cell Dynamics Research Center, Gwangju Institute of Science and Technology, 123 Cheomdangwagi-ro, Buk-gu, Gwangju, 61005 Republic of Korea; Cell Dynamics Research Center and School of Life Science, Gwangju Institute of Science and Technology, 123 Cheomdangwagi-ro, Buk-gu, Gwangju, 61005 Republic of Korea; Department of Orthopedic Surgery, Chonnam National University Medical School and Hospital, 42 Jebong-ro, Dong-gu, Gwangju, 61469 Republic of Korea; Department of Pharmacology and Dental Therapeutics, School of Dentistry, Chonnam National University, 77 Yongbong-ro, Buk-gu, Gwangju, 61186 Republic of Korea; Research Center for Biomineralization Disorders, School of Dentistry, Chonnam National University, 77 Yongbong-ro, Buk-gu, Gwangju, 61186 Republic of Korea

**Keywords:** Rheumatoid arthritis, Cartilage destruction, Chemokine, HIF-2α

## Abstract

**Introduction:**

Pannus formation and resulting cartilage destruction during rheumatoid arthritis (RA) depends on the migration of synoviocytes to cartilage tissue. Here, we focused on the role of hypoxia-inducible factor (HIF)-2α-induced chemokines by chondrocytes in the regulation of fibroblast-like synoviocyte (FLS) migration into the cartilage-pannus interface and cartilage erosion.

**Methods:**

Collagen-induced arthritis (CIA), K/BxN serum transfer, and tumor necrosis factor-α transgenic mice were used as experimental RA models. Expression patterns of HIF-2α and chemokines were determined via immunostaining, Western blotting and RT-PCR. FLS motility was evaluated using transwell migration and invasion assays. The specific role of HIF-2α was determined via local deletion of HIF-2α in joint tissues or using conditional knockout (KO) mice. Cartilage destruction, synovitis and pannus formation were assessed via histological analysis.

**Results:**

HIF-2α and various chemokines were markedly upregulated in degenerating cartilage and pannus of RA joints. HIF-2α induced chemokine expression by chondrocytes in both primary culture and cartilage tissue. HIF-2α -induced chemokines by chondrocytes regulated the migration and invasion of FLS. Local deletion of HIF-2α in joint tissues inhibited pannus formation adjacent to cartilage tissue and cartilage destruction caused by K/BxN serum transfer. Furthermore, conditional knockout of HIF-2α in cartilage blocked pannus formation in adjacent cartilage but not bone tissue, along with inhibition of cartilage erosion caused by K/BxN serum transfer.

**Conclusion:**

Our findings suggest that chemokines induced by IL-1β or HIF-2α in chondrocytes regulate pannus expansion by stimulating FLS migration and invasion, leading to cartilage erosion during RA pathogenesis.

## Introduction

Rheumatoid arthritis (RA) is a chronic autoimmune disease causing synovial inflammation, bone erosion, and cartilage destruction. The inflammatory process initially affects a single joint, but the disease usually progresses to affect nearly all joints [1]. RA is characterized by abnormal synovial hyperplasia caused by increased proliferation and activation of synoviocytes in inflamed synovia [2, 3]. The pannus, an aggressive front of hyperplastic synovium, invades and destroys mineralized cartilage and bone [4–6]. Migration and invasion of activated fibroblast-like synoviocytes (FLS) into cartilage and bone are critical events during invasive pannus formation in RA synovium [4–6]. However, little is currently known on the molecular mechanisms underlying migration and invasion of activated FLS and formation of pannus.

FLS in the inflamed synovium produce numerous catabolic regulators, including chemokines, cytokines, and matrix metalloproteases (MMPs), which are directly implicated in joint destruction [7, 8]. Production of these factors, in addition to cell–cell interactions, is crucial for progression of RA [8, 9]. For instance, FLS produce CX_3_CL1, which attracts T cells to the joint [10]. T cells promote tumor necrosis factor alpha (TNFα) production, in turn causing FLS proliferation [10]. FLS in inflamed synovium also produce several other chemokines, including CXCL8, CXCL9, CXCL10, CXCL12, CCL2, and CCL3 [7, 11–13]. Chemokines are key players in inducing directed chemotaxis in nearby responsive cells and are classified into four subfamilies, specifically CXC, CC, CX_3_C, and XC [14]. These signaling proteins are essential for leukocyte trafficking during chronic inflammatory diseases, such as RA, atherosclerosis, and adipose inflammation [15], and additionally stimulate migration, proliferation, and MMP activation in FLS during RA pathogenesis [16]. Chemokines are also produced by chondrocytes in cartilage tissues of RA joints [17–19], but the potential role of hypoxia-inducible factor (HIF)-2α-induced chemokines in regulation of RA pathogenesis remains to be explored.

Previously, we demonstrated that HIF-2α (encoded by *Epas1*), a transcription factor regulated by oxygen tension [20], acts as an essential catabolic regulator of osteoarthritis (OA) by modulating the expression of various catabolic factors, including MMPs, in chondrocytes [21]. HIF-2α additionally regulates inflammatory RA by modulating angiogenesis, various functions of FLS, and interleukin (IL)-6-dependent T-helper (T_H_) 17 cell differentiation [7]. We initially demonstrated that HIF-2α modulates the expression of various chemokines in both chondrocytes and FLS. Since no links have been established between HIF-2α-induced chemokines, pannus formation, and cartilage erosion, we focused on the potential role of chondrocyte-derived chemokines in FLS migration and invasion during RA pathogenesis. Here, we examined the hypothesis that HIF-2α-induced chemokine production regulates interactions of chondrocytes with FLS, resulting in formation and invasion of pannus into cartilage tissue and consequently cartilage erosion in RA-affected joint tissues.

## Methods

### Mice and experimental arthritis

Male DBA/1 J, C57BL/6, *Epas1*^+/−^, *Epas1*^fl/fl^, TNFα transgenic (TG), and K/BxN mice were used for experimental RA studies. *Epas1*^+/−^ C57BL/6 mice were backcrossed against the DBA/1 J strain for eight generations to generate *Epas1*^+/−^ DBA/1 J [7]. TNFα TG and K/BxN mice were obtained from Taconic Farms (Hudson, NY, USA) and Jackson Laboratory (Bar Harbor, ME, USA), respectively. Mice were housed in specific pathogen-free barrier facilities and used in accordance with protocols approved by the Animal Care and Ethics Committees of Chonnam National University and Gwangju Institute of Science and Technology, Gwangju, Republic of Korea. Collagen-induced arthritis (CIA), K/BxN serum transfer, and TNFα TG mice were employed as experimental RA models. CIA was induced in DBA/1 J mice using a previously described standard protocol [7]. For K/BxN serum transfer, K/BxN and control sera were collected from arthritic K/BxN and nonarthritic BxN mice, respectively. K/BxN or control serum (200 μl) was administered intraperitoneally on days 0 and 2, and mice sacrificed 14 days after serum transfer [22]. TNFα TG mice (20 weeks old, heterozygous) were used as a model for spontaneous inflammatory arthritis, with non-TG wild-type (WT) littermates as controls [23].

### Histological analysis of joint tissue and immunostaining

Mouse joint tissues were decalcified with 0.5 M ethylenediamine tetraacetic acid (EDTA, pH 8.0) for 2 weeks, embedded in paraffin, and sectioned at 5 μm thickness. Synovitis was evaluated via hematoxylin and eosin staining of joint sections, and synovial inflammation (grade 0–4) scored as described previously [7]. The pannus in joint tissues adjacent to cartilage and bone was visualized via hematoxylin/safranin-O staining, and pannus formation scored (grades 0–4) as described previously [7]. Cartilage destruction was visualized and scored using Mankin’s method [7]. HIF-2α in joint sections was immunostained with rabbit anti-HIF-2α (Santa Cruz Biotechnology, Dallas, TX, USA) antibody. For double immunofluorescence labeling in joint sections, rabbit anti-HIF-2α (Novus Biologicals, Littleton, CO, USA), rabbit anti-CXCL1 (Abcam, Cambridge, UK), goat anti-CXCL2, and goat anti-CCL5 (R&D Systems, Minneapolis, MN, USA) primary antibodies were used, followed by incubation with Alexa Fluor 594 goat anti-rabbit IgG or Alexa Fluor 488 goat anti-mouse IgG-conjugated secondary antibody (Thermo Fisher Scientific, Waltham, MA, USA).

### FLS and articular chondrocyte culture and conditioned medium preparation

FLS were isolated from joint synovium of WT and *Epas1*^+/−^ mice according to previously described protocols [7]. FLS between passages 4 and 8 were used for further analysis. Flow cytometry using antibodies against the fibroblast marker, CD90, and the macrophage marker, CD14 (Abcam), led to the identification of pure FLS (>90 % CD90^+^/<1 % CD14^+^). Chondrocytes were isolated from cartilage tissue by digestion with 0.2 % collagenase type II, as described previously [21, 24], and cells on culture day 3 used for further treatments. To prepare conditioned medium (CM), chondrocytes or FLS were treated with IL-1β or infected with 800 multiplicity of infection (MOI) of empty adenovirus (Ad-C) or adenovirus expressing HIF-2α (Ad-*Epas1*) for 24 hours in the absence of serum.

### RNA isolation, RT-PCR, quantitative RT-PCR, and western blotting

Total RNA was extracted from mouse articular chondrocytes and knee joint cartilage using TRI reagent. For isolation of RNA from knee joints, cartilage tissues were sliced using a surgical blade and RNA isolated using the Purelink RNA mini kit (Thermo Fisher Scientific). RNA was reverse-transcribed and cDNA amplified using PCR. Transcript levels were quantified with quantitative real time-PCR (qRT-PCR) performed using SYBR Premix Ex Taq™ (TaKaRa Bio Inc., Shiga, Japan). All qRT-PCR reactions were performed in duplicate, and threshold cycle values from target genes normalized to that of glyceraldehyde-3-phosphate dehydrogenase (GAPDH). Primer sequences for PCR were described in an earlier report [7]. Western blot analysis was performed to detect secreted CXCL1, CXCL2, and CCL5 in CM using the following antibodies: rabbit anti-CXCL1 (Abcam), goat anti-CXCL2 (R&D Systems), and goat anti-CCL5 (R&D Systems).

### Chromatin immunoprecipitation assay

Chromatin immunoprecipitation (ChIP) assays of mouse articular chondrocytes infected with Ad-*Epas1* (800 MOI) were performed using a Magna ChIP™ kit (EMD Millipore, Billerica, MA, USA) [25]. Primers for the ChIP assay were designed to amplify HIF responsive element (HRE)-containing promoter regions of genes of the indicated chemokines. Sequences of primers for the ChIP assay were as follows: *Cxcl1* #1-F, 5′-CAGATGAGAAACATACTTGAGG-3′ and #1-R, 5′-GGAGAACTGGAGCTATCATG-3′; *Cxcl1* #2-F, 5′-AGCATTCTAAAATAAACAGGG-3′ and #2-R, 5′-GGCAGATTAACGCATTCTT-3′; *Cxcl1* #3-F, 5′-TGGGATAAGAGAGGGTAGATG-3′ and #3-R, 5′-GACGTGCTTCGCTGGAC-3′; *Cxcl2*-F, 5′-CCAAACTGTTAGGTCTCCAC-3′ and *Cxcl2*-R, 5′-CTGAGTGGGTTGGGGAC-3′; *Cxcl5* #1-F, 5′-CCAACCCACTCAGCTTAGG-3′ and #1-R, 5′-GGCGCTAGGCTGAAGTG-3′; *Cxcl5* #2-F, 5′-TGTGTATGTCTGCTTATCTGTCT-3′ and #2-R, 5′-ACACTATTGCTGACACCTGG-3′; *Cxcl10* #1-F, 5′-CTGAACCAAGGATCTGCTC-3′ and #1-R, 5′-TGTACAAGTTCTCAGTCAAGATG-3′; *Cxcl10* #2-F, 5′-CTTCCGGCTTCTGTTCTG-3′ and #2-R, 5′-CTAAGTCAGGTTCTAACCATGG-3′; and *Ccl5*-F, 5′-CCAATTTCAGACCCTACC-3′ and *Ccl5*-R, 5′-GGCTCAGAACACATTGCA-3′.

### Cell migration and invasion assay

For the transwell migration and invasion assay, FLS from WT (*Epas1*^+/+^) or *Epas1*^+/−^ mice (C57BL/6) were seeded on membranes of inserts with 8.0 μm pores (Corning Costar, Corning, NY, USA ) and the lower chambers filled with CM, which served as the chemoattractant. Serum-free CM was prepared from primary cultures of chondrocytes or FLS isolated from WT or *Epas1*^+/−^ mice (C57BL/6). For neutralization of chemokines, 0.5 μg anti-IgG (control), anti-CXCL2, or anti-CCL5 (R&D Systems) were added to the lower chamber. For the invasion assay, inserts of the transwell were precoated with matrigel (BD Bioscience, San Jose, CA, USA). After 24 hours of incubation, transwell inserts were fixed with 4 % paraformaldehyde for 10 minutes and stained with 0.05 % crystal violet. Cells on the bottom layer were captured in five randomly selected fields at 200× magnification using a light microscope and quantified with Image J software (NIH, shareware) .

### Microarray analysis

Mouse articular chondrocytes and FLS were infected with Ad-*Epas1* or empty virus at an MOI of 800 for 24 hours. Total RNA was extracted from mouse articular chondrocytes or mouse FLS cells using TRI reagent (Molecular Research Center, Cincinnati, OH, USA). Three replicates for each cell type were isolated and processed. RNA of mouse articular chondrocytes was analyzed using Agilent microarrays (Agilent Mouse Whole Genome 4 × 44 K Microarray), in accordance with the Agilent protocol (Genomic Tree Inc., Daejeon, Korea ). RNA of mouse FLS was analyzed using Affymetrix GeneChip arrays (Affymatrix GeneChip Mouse Gene 2.0 ST Array) using the Affymetrix protocol (DNALINK Inc., Seoul, Korea). All microarray raw data are available through the GEO database [GEO:GSE73658, GEO:GSE73659].

### Statistical analysis

The nonparametric Mann–Whitney *U* test was used for analysis of data based on an ordinal grading system, such as synovitis, pannus, and Mankin scores. Data obtained with qRT-PCR assays, cell migration and invasion, and HIF-2α-positive cells were initially tested for conformation to normal distribution using the Shapiro–Wilk test and subsequently analyzed with Student’s *t* test (pair-wise comparisons) or analysis of variance (ANOVA) with post hoc tests (multicomparison), as appropriate. Significance was accepted at the 0.05 level of probability (*P* <0.05).

## Results

### HIF-2α is upregulated in both cartilage and pannus of the experimental mouse RA joint

**Fig. 1:**
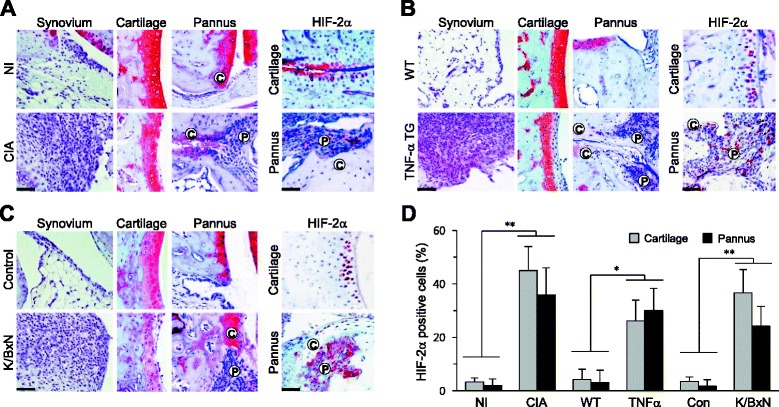
HIF-2α is upregulated in cartilage and pannus of mouse experimental RA joint tissue. **a** *Left* Representative images of synovium, cartilage, and pannus in ankle joints of DBA/1 J mice either immunized with type II collagen (CIA) or not immunized (*NI*) (*n* = 15). *Right* Representative HIF-2α immunostaining in cartilage and pannus of ankle joints of DBA/1 J mice (*n* = 10). **b** *Left* Representative images of synovium, cartilage, and pannus in ankle joints of 20-week-old TNFα TG mice (*n* = 8). *Right* Representative HIF-2α immunostaining in cartilage and pannus of ankle joints of TNFα TG mice (*n* = 8). **c** *Left* Representative images of synovium, cartilage, and pannus in ankle joints of mice transferred with K/BxN serum or control serum (*n* = 12). *Right* Representative HIF-2α immunostaining in cartilage and pannus (*n* = 7). **d** Relative expression levels of HIF-2α in cartilage and pannus. HIF-2α-positive cells were counted in the indicated RA mouse model. Values presented as mean ± standard error of the mean (*P <0.001, **P <0.0005). Scale bar: 50 μm. *C* cartilage, *CIA* collagen-induced arthritis, *CON* control, *HIF* hypoxia-inducible factor, *P* pannus, *TG* transgenic, *TNFα* tumor necrosis factor alpha, *WT* wild type

To elucidate the possible links and regulatory mechanisms between pannus formation and cartilage erosion, we initially examined HIF-2α expression levels in chondrocytes from cartilage and FLS in pannus of inflamed joint tissues. CIA [7], K/BxN serum transfer [22], and TNFα TG mice [23] were employed as inflammatory arthritis models. CIA mice displayed severe synovitis, pannus formation, and cartilage erosion in the ankle joint, along with a marked increase in HIF-2α protein levels in chondrocytes of damaged cartilage and FLS in pannus adjacent to cartilage tissue (Fig. 1a). Similarly, TNFα TG mice (20 weeks old) exhibited synovitis, pannus formation, and cartilage erosion with increased HIF-2α levels in chondrocytes and FLS of cartilage and pannus, respectively (Fig. 1b). Additionally, HIF-2α was markedly upregulated in cartilage and pannus in the K/BxN serum transfer model, with severe synovitis and cartilage erosion (Fig. 1c). Quantitation of HIF-2α-positive cells in cartilage and pannus of RA-affected joints revealed significantly increased numbers of chondrocytes and FLS in all the models of inflammatory arthritis examined (Fig. 1d).

### HIF-2α upregulates chemokine expression in both FLS and chondrocytes

**Fig. 2:**
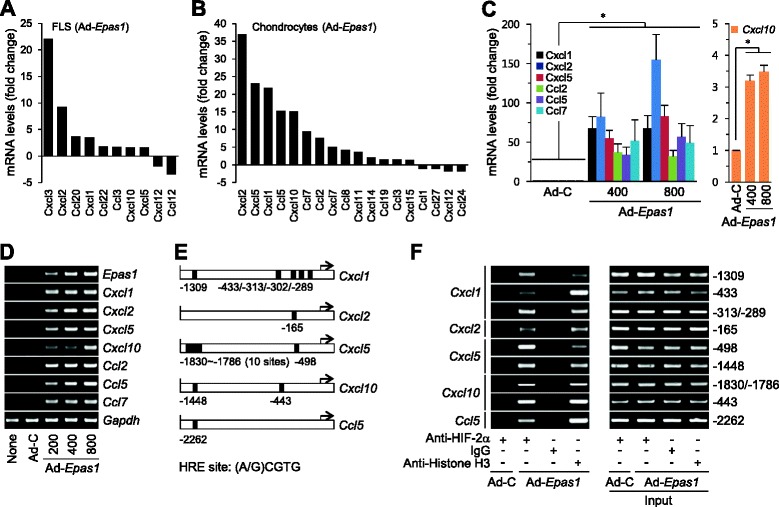
HIF-2α upregulates chemokine expression in FLS and chondrocytes. Microarray analysis of chemokines in primary culture mouse FLS **a** or articular chondrocytes **b** infected with 800 MOI of Ad-C and Ad-*Epas1*. Relative mRNA levels against Ad-C are shown (*n* = 3). **c**, **d** Primary cultures of mouse articular chondrocytes were left untreated (*None*) or infected with Ad-C (800 MOI) and the indicated amounts (MOI) of Ad-*Epsa1* for 24 hours. Levels of indicated mRNA were determined with qRT-PCR **c** and RT-PCR **d** analyses. Transcripts of the indicated genes were examined using RT-PCR **d** and quantified with qRT-PCR **c** (*n* = 5). **e** HIF-2α binding sites in promoters of indicated chemokines. ChIP assays in chondrocytes infected with 800 MOI Ad-C or Ad-*Epas1*. The histone H3 antibody was used as the positive control and IgG as a negative control. **f** The indicated HRE sites in each chemokine promoter were amplified from immunoprecipitated DNA (*left*) and pre-immunoprecipitated DNA (*right*; input). Values presented as mean ± standard error of the mean (*P <0.005). *FLS* fibroblast-like synoviocytes, *HIF* hypoxia-inducible factor, *HRE* hypoxia-inducible factor responsive element

Next, we examined whether HIF-2α regulates chemokine expression in chondrocytes and FLS. Microarray analyses of HIF-2α-overexpressing FLS revealed markedly increased mRNA levels of CXCL3 (22.1-fold), CXCL2 (9.3-fold), CCL20 (3.7-fold), CXCL1 (3.5-fold), and CXCL5 (1.6-fold) (Fig. 2a). HIF-2α overexpression in chondrocytes also led to a marked increase in the mRNA levels of CXCL2 (37.0-fold), CXCL5 (23.0-fold), CXCL1 (21.9-fold), CCL5 (15.3-fold), CXCL10 (15.2-fold), CCL7 (9.4-fold), CCL2 (7.6-fold), CXCL7 (5.1-fold), CCL8 (4.2-fold), and CXCL11 (3.7-fold) (Fig. 2b). Upregulation of chemokines by HIF-2α in primary culture chondrocytes was further verified with RT-PCR and qRT-PCR (Fig. 2c, d). Using the ChIP assay, we additionally investigated whether these chemokines are direct target genes of HIF-2α in mouse chondrocytes. Promoters of *Cxcl1*, *Cxcl2*, *Cxcl5*, *Cxcl10*, *Ccl2*, and *Ccl5*, but not *Ccl2* and *Ccl7*, contain one or more hypoxia response element (HRE) motifs (−(A/G)CGTG–) (Fig. 2e). ChIP data revealed direct binding of HIF-2α to promoters of *Cxcl1*, *Cxcl2*, *Cxcl5*, *Cxcl10*, and *Ccl5* in primary culture chondrocytes (Fig. 2f).

**Fig. 3:**
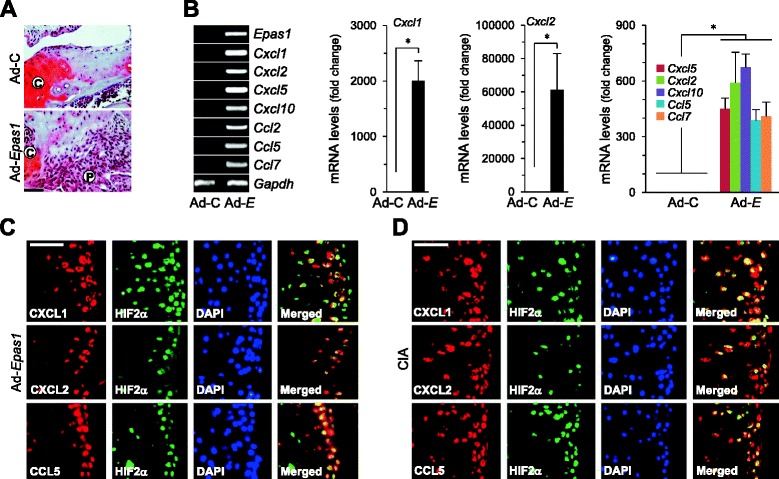
HIF-2α upregulates chemokine expression in RA cartilage tissue. **a**, **b** Empty virus (Ad-C) or Ad-*Epas1* (1 × 10^9^ plaque-forming unit ) was injected into mouse knee joints. Representative hematoxylin/safranin O-stained images in cartilage and pannus regions **a**. Results of RT-PCR and qRT-PCR analysis of the indicated genes in cartilage tissue **b** (*n* = 4). **c**, **d** Typical immunofluorescence microscopy images of DAPI, HIF-2α, CXCL1, CXCL2, and CCL5 in cartilage tissue of mice after IA injection with Ad-*Epas1* **c** or CIA **d**. Values presented as mean ± standard error of the mean (*P <0.01). Scale bar: 50 μm. *C* cartilage, *CIA* collagen-induced arthritis, *DAPI* 4′,6-diamidino-2-phenylindole, *HIF* hypoxia-inducible factor, *P* pannus

Next, we examined whether HIF-2α regulates the expression of chemokines in mouse cartilage tissue. Consistent with previous findings [7], intra-articular (IA) injection of Ad-*Epas1* promoted pannus formation (Fig. 3a). RT-PCR and qRT-PCR analyses of cartilage tissue revealed that HIF-2α induces a significant increase in mRNA levels of the chemokines examined (Fig. 3b). Furthermore, HIF-2α-overexpressing chondrocytes in cartilage tissue of mice IA injected with Ad-*Epas1* showed increased levels of CXCL1, CXCL2, and CCL5 proteins, which co-localized with HIF-2α (Fig. 3c). Similarly, CXCL1, CXCL2, and CCL5 proteins were increased in HIF-2α-overexpressing chondrocytes in cartilage tissue of CIA mice and co-localized with HIF-2α (Fig. 3d). Our results collectively indicate that HIF-2α upregulates chemokines in both primary culture chondrocytes and cartilage tissue.

**Fig. 4:**
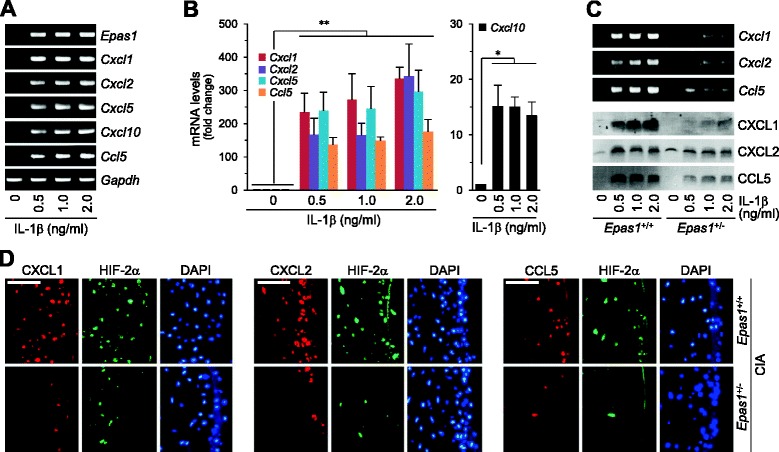
HIF-2α mediates IL-1β-induced chemokine expression in chondrocytes. **a** RT-PCR and **b** qRT-PCR analyses of mRNA in chondrocytes treated with IL-1β for 24 hours (*n* = 5). **c** RT-PCR and western blot analyses of the indicated molecules in primary cultures of chondrocytes isolated from WT (*Epas1* ^+/+^) or *Epas1* ^+/−^ mice treated with IL-1β for 24 hours. RT-PCR was carried out to detect transcript levels of *Epas1*, *Cxcl1*, *Cxcl2*, and *Ccl5*. Secreted chemokine proteins were examined via western blot. **d** Immunostaining of HIF-2α, CXCL1, CXCL2, and CCL5 in cartilage tissue of WT and *Epas1* ^+/−^ DBA1/J mice with CIA. Values presented as mean ± standard error of the mean (*P <0.05, **P <0.01). Scale bar: 50 μm. *CIA* collagen-induced arthritis, *DAPI* 4′,6-diamidino-2-phenylindole, *HIF* hypoxia-inducible factor, *IL* interleukin

IL-1β is one of the major proinflammatory cytokines regulating RA pathogenesis [26]. IL-1β also transcriptionally upregulates HIF-2α in FLS and chondrocytes [7, 21]. Accordingly, we examined whether IL-1β stimulates chemokine expression via the HIF-2α pathway in chondrocytes. IL-1β enhanced chemokine expression in WT chondrocytes (Fig. 4a, b). This upregulation was markedly inhibited in *Epas1*^+/−^ chondrocytes (Fig. 4c). We previously showed that deletion of one allele of *Epas1* significantly inhibits the hallmarks of RA, including synovitis, cartilage erosion, and pannus formation [7]. Consistently, *Epas1*^+/−^ DBA/1 J mice under CIA conditions showed marked reduction of CXCL1, CXCL2, and CCL5 expression, compared with their corresponding WT littermates (Fig. 4d). These findings suggest that regulation of chemokines in RA cartilage is controlled by HIF-2α.

### HIF-2α-induced chemokines by chondrocytes regulate FLS migration and invasion

**Fig. 5:**
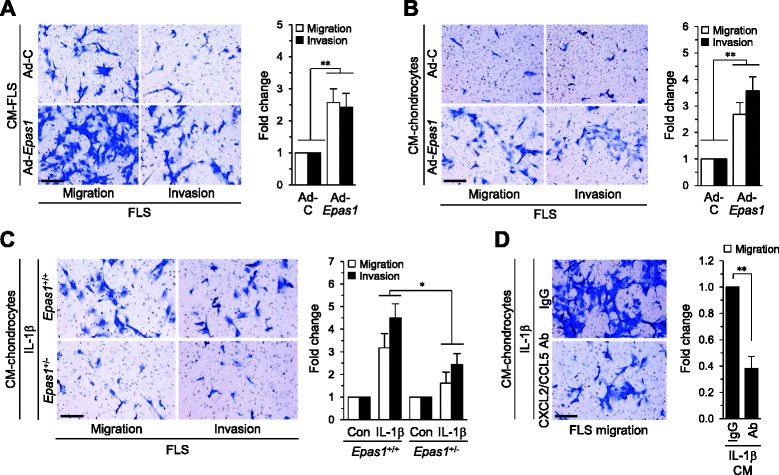
HIF-2α-induced chemokine expression in chondrocytes causes FLS migration and invasion. **a** Migration and invasion of FLS treated (12 hours) with conditioned medium (*CM*) prepared from FLS infected with 800 MOI Ad-C or Ad-*Epas1*. Relative fold changes are presented (*n* = 6). **b** Migration and invasion of FLS treated (12 hours) with CM prepared from chondrocytes infected with 800 MOI Ad-C or Ad-*Epas1*. Relative fold changes are presented (*n* = 6). **c** Migration and invasion of FLS treated (12 hours) with CM prepared from WT (*Epas1* ^+/+^) or *Epas1* ^+/−^ chondrocytes treated with IL-1β (2 ng/ml, 24 hours) (*n* = 4). **d** Migration of FLS treated with CM prepared from chondrocytes exposed to IL-1β (2 ng/ml, 24 hours) in the presence of IgG or neutralizing antibodies against CXCL2 and CCL5. Values presented as mean ± standard error of the mean (*P <0.05, **P <0.005). Scale bar: 50 μm. *Ab* antibody, *CON* control, *FLS* fibroblast-like synoviocytes, *IL* interleukin

FLS, a major cell type in pannus tissue, migrate, attach to, invade, and destroy cartilage tissue by secreting proteases within the RA joints [4]. Since a major function of chemokines is recruitment of adjacent cells as a chemoattractant, we used an in vitro modified Boyden chamber assay to further examine whether increased expression of chemokines by HIF-2α in FLS and chondrocytes plays a role in FLS migration and invasion. Our data initially confirmed that CM from FLS infected with Ad-*Epas1* stimulates both migration and invasion of FLS (Fig. 5a). Next, we examined whether HIF-2α-induced chemokines by chondrocytes also regulate migration and invasion of FLS. Notably, CM from Ad-*Epas1*-infected chondrocytes promoted both migration and invasion of FLS (Fig. 5b). The correlation between chondrocyte-originating HIF-2α and chemokine function in FLS migration and invasion was further validated. CM from chondrocytes treated with IL-1β stimulated the migration and invasion of WT (*Epas1*^+/+^) FLS (Fig. 5c). However, *Epas1*^+/−^ FLS exhibited significantly reduced migration and invasion in the presence of IL-1β-treated chondrocyte CM (Fig. 5c). Furthermore, addition of anti-CXCL2 and anti-CCL5 antibodies to CM of IL-1β-treated chondrocytes blocked FLS migration (Fig. 5d). Based on the results, we propose that HIF-2α-induced chemokines by chondrocytes regulate FLS migration and invasion.

### Cartilage-specific knockdown of HIF-2α inhibits FLS migration at the cartilage–pannus interface

**Fig. 6:**
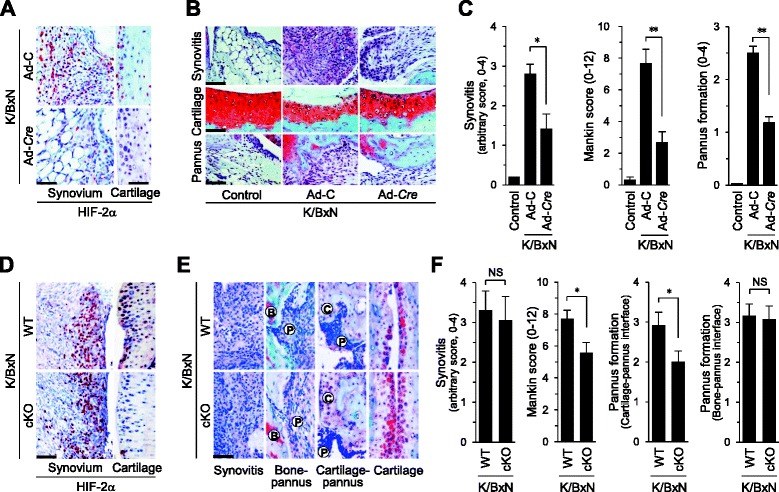
Knockdown of HIF-2α in joint tissues inhibits cartilage destruction at the cartilage–pannus interface. **a**–**c** *Epas1* ^fl/fl^ mice were IA-injected with Ad-C or Ad-*Cre* (1 × 10^9^ plaque-forming unit ) to locally delete HIF-2α in joint tissue. K/BxN serum was transferred into mice to induce experimental RA. Representative images of HIF-2α immunostaining in the synovium and cartilage of ankle joints (*n* = 8) **a**. Detection **b** and quantitation **c** of synovitis, cartilage destruction (Mankin scores), and pannus formation (*n* = 10). **d**–**f** K/BxN serum was transferred to *Epas1* ^fl/fl^ (WT) or *Epas1* ^fl/fl^;*Col2a1*-*cre* mice. Representative images of HIF-2α immunostaining in the synovium and cartilage of ankle joints **d**. Detection **e** and quantitation **f** of synovitis, cartilage destruction (Mankin scores), and pannus formation (*n* = 5). Values presented as mean ± standard error of the mean (*P <0.01, **P <0.001; *NS* not significant). Scale bar: 50 μm. *B* bone, *C* cartilage, *cKO* conditional knockout, *HIF* hypoxia-inducible factor, *P* pannus, *WT* wild type

Finally, we elucidated the in vivo significance of HIF-2α-derived chemokine production in chondrocytes during RA cartilage destruction. For this purpose, HIF-2α was locally downregulated in joint tissues via IA injection of Ad-*Cre* in *Epas1*^fl/fl^ mice and experimental RA induced by K/BxN serum transfer. Immunostaining in synovium and cartilage revealed that Ad-*Cre* injection effectively reduced the levels of HIF-2α induced by K/BxN serum transfer (Fig. 6a). Local deletion of *Epas1* in joint tissues significantly suppressed all the hallmarks of RA, including synovitis and synovial hyperplasia, pannus formation and invasion into calcified cartilage and bone, and cartilage destruction (Fig. 6b, c). To elucidate the role of HIF-2α-regulated chemokines in cartilage, we used chondrocyte-specific conditional knockout (cKO) mice (*Epas1*^fl/fl^;*Col2a1*-*cre*) in a K/BxN serum transfer model. In cKO mice, K/BxN serum transfer did not cause upregulation of HIF-2α in cartilage whereas HIF-2α was upregulated in synovial tissue (Fig. 6d). Consistently, *Epas1*^fl/fl^;*Col2a1*-*cre* mice exhibited significantly reduced cartilage erosion and pannus formation adjacent to cartilage tissue after K/BxN serum transfer, compared with *Epas1*^fl/fl^ mice (Fig. 6e, f). However, K/BxN serum transfer caused a similar degree of synovitis and bone loss at the bone–pannus junction (Fig. 6e, f), indicating that HIF-2α knockout in cartilage inhibits pannus expansion and FLS migration in RA. Our collective findings suggest that HIF-2α-stimulated chemokines in chondrocytes regulate FLS motility, resulting in pannus invasion into the cartilage during RA pathogenesis.

## Discussion

Our current results suggest that RA-triggering factors, such as IL-1β and HIF-2α, enhance the expression of several chemokines in chondrocytes, including *Cxcl1*, *Cxcl2*, *Cxcl5*, *Cxcl10*, *Ccl2*, *Ccl5*, and *Ccl7.* These HIF-2α-induced chemokines in chondrocytes stimulate migration and invasion of FLS, leading to pannus expansion adjacent to cartilage tissue of RA joints. Functional blockage of HIF-2α-induced chemokines, such as *Cxcl2* and *Ccl5*, ameliorated FLS motility. Moreover, *Cxcl1*, *Cxcl2*, *Cxcl5*, *Cxcl10*, and *Ccl5* were identified as direct target genes of HIF-2α in chondrocytes. Local deletion of HIF-2α in joint tissues or conditional knockout in cartilage tissue inhibited pannus formation adjacent to cartilage tissue and cartilage destruction caused by K/BxN serum transfer, demonstrating the significance of chondrocyte-originated chemokines in pannus expansion and cartilage destruction during RA pathogenesis.

Formation of invasive pannus, a thickened layer of synovial tissue that erodes cartilage and bone, is the pathologic hallmark of RA [2, 3]. FLS is a main effector cell type in the progression of RA, promoting pannus formation with cartilage erosion and progressive joint destruction by secreting diverse cytokines, chemokines, and MMPs [5, 8, 9]. Here, we demonstrated upregulation of HIF-2α in chondrocytes from damaged cartilage and FLS in the pannus region of various RA mouse models, including CIA, TNFα TG, and K/BxN serum transfer. To elucidate the potential functions of HIF-2α, we screened the gene expression profiles in HIF-2α-overexpressing chondrocytes and FLS via microarray analysis. Functional enrichment analysis of microarray data revealed upregulation of genes associated with RA pathogenesis, such as those involved in extracellular matrix (ECM) remodeling and inflammatory response-related, cell migration-related and invasion-related processes (data not shown). Among these, we focused on chemokines expressed in both chondrocytes and FLS. Chemokines regulate chemotaxis in nearby responsive cells and are implicated in chronic inflammatory diseases, such as RA, atherosclerosis, and adipose inflammation [14].

Release of chemoattractants by FLS signals various immune cells to migrate into the RA synovium [4]. Chemokines, such as CX_3_CL1, CCL2, and CXCL10, and their receptors, CCR2, CCR5, and CXCR3, regulate FLS migration or invasion during RA pathogenesis [16, 27, 28]. In addition to FLS, chondrocytes produce various chemokines and related catabolic factors during cartilage destruction. For instance, human OA chondrocytes generate various chemokines (CXCL1, CCL2, and CCL5) and chemokine receptors (CCR1, CCR2, CCR3, CCR5, CXCR1, and CXCR2) by proinflammatory cytokines [17–19]. CXCR1/CXCR2 production by human OA chondrocytes stimulates MMP3 release [19], and constitutively expressed CXCR2 in normal chondrocytes maintains cartilage homeostasis [29]. However, the functions and regulatory mechanisms of HIF-2α-induced chemokines in RA pathogenesis are yet to be elucidated. Our current results clearly demonstrate that HIF-2α upregulates various chemokines as direct target genes in primary culture chondrocytes. Additionally, upregulated HIF-2α in cartilage tissue stimulates chemokine expression in chondrocytes. We detected three CCL (CCL2, CCL5, and CCL7) and four CXCL chemokine families (CXCL1, CXCL2, CCXCL5, and CXCL10) that were highly upregulated by IL-1β or HIF-2α. Among these, *Cxcl1*, *Cxcl2*, *Cxcl5*, *Cxcl10*, and *Ccl5*, but not *Ccl2* and *Ccl7*, were identified as direct target genes of HIF-2α. *Ccl2* and *Ccl7* genes may be regulated indirectly or independently of HIF-2α. NF-κB is a potential transcription factor responsible, since the promoter region contains a NF-κB binding site and IL-1β-induced HIF-2α upregulation is controlled by NF-κB signaling [21]. Furthermore, our data show that HIF-2α-induced chemokines by chondrocytes stimulate in vitro migration and invasion of FLS. The observation that addition of blocking antibodies against CXCL2 and CCL5 to CM of chondrocytes abrogates FLS migration clearly demonstrates an essential role of HIF-2α-induced chemokines by chondrocytes in FLS motility. Although chemokines are produced by both chondrocytes and FLS, it remains to be established whether HIF-2α-induced chemokines by chondrocytes act in a manner complementary to or redundant with those produced by FLS in the development and pathogenesis of experimental RA. Different chemokine subtypes are upregulated in FLS and chondrocytes. For instance, CXCL3 and CXCL2 are the most highly upregulated in Ad-*Epas1*-infected FLS, while CXCL2, CXCL5, CXCL1, and CCL5 are significantly upregulated by HIF-2α-overexpressing chondrocytes. Among these, CXCL2 expression was the most upregulated in Ad-*Epas1*-infected chondrocytes. Chondrocyte-derived CXCL2 appears to be a major chemoattractant triggering FLS migration, based on data showing that blockage of CXCL2 with specific antibodies inhibits FLS migration in IL-1β-treated CM. All chemokines exert biological effects by stimulating G protein-linked receptors [14]. Microarray analysis revealed the presence of CXCR2 (for CXCL1, CXCL2, and CXCL5), CXCR3 (for CXCL10), and CCR5 (for CCL5) [14] in both mouse FLS and articular chondrocytes, although the expression levels of these receptors were not affected by HIF-2α overexpression (data not shown).

Chondrocytes in cartilage tissue are embedded in cartilage matrix, raising the question of how chondrocyte-derived chemokines regulate the motility of FLS, which are localized in synovial tissues. One possibility is that chemokines diffuse from the cartilage matrix to affect synovial cells [30]. Although we did not validate this theory in the current study, we were able to demonstrate the significance of chondrocyte-derived chemokines in the expansion of invasive pannus in vivo. Local deletion of HIF-2α in joint tissues via IA injection of Ad-*Cre* in *Epas1*^fl/fl^ mice suppressed pannus formation and cartilage destruction. Furthermore, cKO of HIF-2α in cartilage tissue, which inhibited chemokine production, led to sufficient blockade of pannus formation adjacent to cartilage tissue, but not in bone tissue, resulting in inhibition of cartilage destruction. Cartilage is eroded by breakdown of ECM via the action of several proteases, such as MMPs and cathepsin, during RA. Our group previously showed that stimulation of chondrocytes with IL-1β increases secretion of MMPs in association with marked HIF-2α transcriptional activity [7, 21]. MMPs participate in ECM remodeling and play important roles in the progressive destruction of joints in RA. FLS are considered a major effector cell type involved in cartilage erosion, based on their ability to produce massive amounts of degradative enzymes, such as MMPs. Since both FLS and cartilage are major cell types that produce ECM-degrading enzymes [7, 21], cartilage erosion during RA may be attributable to a multistep and complex process including “FLS attachment to cartilage” and “synthesis of enzymes from FLS and cartilage that degrade the ECM”. This theory is supported by the finding that deletion of HIF-2α in both synovium and cartilage exerts a greater ameliorating effect on cartilage destruction than local deletion in cartilage.

## Conclusions

In summary, HIF-2α is overexpressed in damaged chondrocytes as well as FLS in the pannus region in several experimental RA mouse models, and directly regulates the expression of *Cxcl1*, *Cxcl2*, *Cxcl5*, *Cxcl10*, and *Ccl5*. In addition, we have identified a previously unrecognized role of chondrocyte-derived chemokines and provided a mechanistic explanation for the regulatory processes underlying the invasive properties of FLS. Although our findings from experimental RA mouse models remain to be verified in human RA pathogenesis, we carefully suggest new insights into the effects of chondrocytes on synovial cell motility into the cartilage–pannus interface in RA pathogenesis and highlight the relevance of HIF-2α as a potential novel therapeutic target in RA.

## Abbreviations

ANOVA: Analysis of variance
ChIP: Chromatin immunoprecipitation
CIA: Collagen-induced arthritis
cKO: Conditional knockout
CM: Conditioned medium
ECM: Extracellular matrix
EDTA: Ethylenediamine tetraacetic acid
FLS: Fibroblast-like synoviocytes
GAPDH: Glyceraldehyde-3-phosphate dehydrogenase
HIF: Hypoxia-inducible factor
HRE: Hypoxia-inducible factor responsive element
IA: Intra-articular
IL: Interleukin
MMP: Matrix metalloproteinase
MOI: Multiplicity of infection
OA: Osteoarthritis
qRT-PCR: Quantitative RT-PCR
RA: Rheumatoid arthritis
TG: Transgenic
T_H_: T-helper
TNFα: Tumor necrosis factor alpha
WT: Wild type
